# Tris(5-methyl-3-phenyl-1*H*-pyrazol-1-yl)methane

**DOI:** 10.1107/S1600536808010866

**Published:** 2008-04-23

**Authors:** David J. Harding, Phimphaka Harding, Sarah E. Plant

**Affiliations:** a Molecular Technology Unit Cell, Department of Chemistry, Walailak University, Thasala, Nakorn Si Thammarat 80161, Thailand; b Department of Chemistry, Faculty of Science, University of Sheffield, Brook Hill, Sheffield S3 7HF, England

## Abstract

The first crystal structure of a second-generation tris­(pyrazol­yl)methane, namely the title compound, C_31_H_28_N_6_, is reported. The mol­ecule exhibits a helical conformation with an average twist of 35.1°. In addition, there are C—H⋯π inter­actions of 3.202 (2) Å between the pyrazole C—H group and neighbouring phenyl groups.

## Related literature

For related literature, see: Astley *et al.* (1993 ▶); Fujisawa *et al.* (2004 ▶); Goodman & Bateman (2001 ▶); Ochando *et al.* (1997 ▶); Pettinari & Pettinari (2005 ▶); Reger *et al.* (2000 ▶, 2002 ▶); Riche & Pascard-Billy (1974 ▶); Declercq & Van Meerssche (1984 ▶).
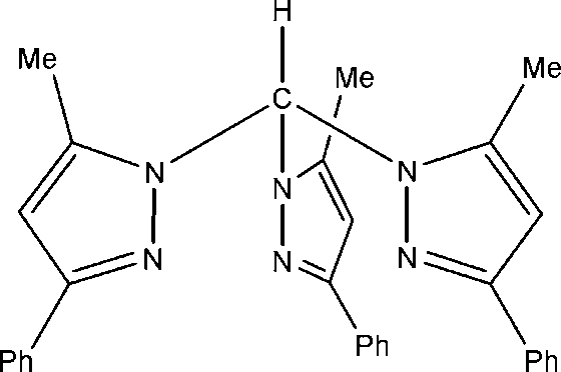


## Experimental

### 

#### Crystal data

C_31_H_28_N_6_
*M* *_r_* = 484.59
Monoclinic, 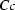

*a* = 6.678 (3) Å
*b* = 21.730 (9) Å
*c* = 17.831 (7) Å
β = 94.922 (7)°
*V* = 2578.2 (19) Å^3^
*Z* = 4
Mo *K*α radiation
μ = 0.08 mm^−1^
*T* = 150 (2) K
0.38 × 0.34 × 0.21 mm

#### Data collection

Bruker SMART CCD area-detector diffractometer
Absorption correction: multi-scan (*SADABS*; Bruker, 1997 ▶) *T*_min_ = 0.972, *T*_max_ = 0.984
9586 measured reflections
2932 independent reflections
2129 reflections with *I* > 2σ(*I*)
*R*_int_ = 0.039

#### Refinement

*R*[*F* ^2^ > 2σ(*F* ^2^)] = 0.041
*wR*(*F* ^2^) = 0.071
*S* = 0.98
2932 reflections
337 parameters
2 restraints
H-atom parameters constrained
Δρ_max_ = 0.12 e Å^−3^
Δρ_min_ = −0.14 e Å^−3^

### 

Data collection: *SMART* (Bruker, 1997 ▶); cell refinement: *SMART*; data reduction: *SAINT* (Bruker, 1997 ▶); program(s) used to solve structure: *SHELXS97* (Sheldrick, 2008 ▶); program(s) used to refine structure: *SHELXL97* (Sheldrick, 2008 ▶); molecular graphics: *SHELXTL* (Sheldrick, 2008 ▶); software used to prepare material for publication: *SHELXTL*.

## Supplementary Material

Crystal structure: contains datablocks I, global. DOI: 10.1107/S1600536808010866/rk2085sup1.cif

Structure factors: contains datablocks I. DOI: 10.1107/S1600536808010866/rk2085Isup2.hkl

Additional supplementary materials:  crystallographic information; 3D view; checkCIF report

## Figures and Tables

**Fig. 1.**
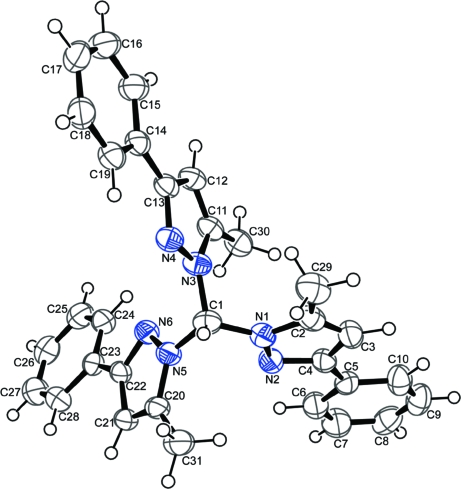
The molecular structure of the title compound showing the atom–labelling scheme. Displacement ellipsoids are drawn at the 50% probability level. H atoms are presented as a spheres of arbitrary radius.

**Fig. 2.**
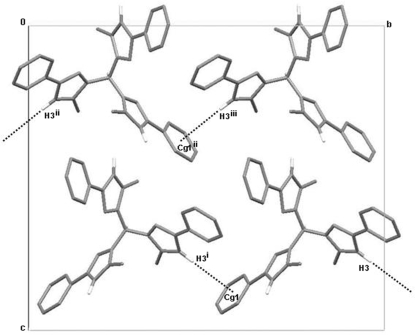
The molecular packing showing the C—H···π interactions in two adjacent chains. Only selected H atoms are shown and labelled for clarity. Symmetry codes: (i) x+1/2, y-1/2, z; (ii) x-1, -y+1, z-1/2; (iii) x-1/2, -y+3/2, z-1/2.

**Fig. 3.**
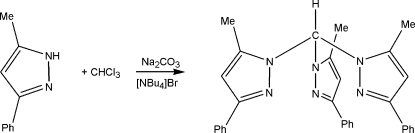
A schematic diagram of formation title compound.

## supplementary crystallographic information

### Comment

*Tris*–(pyrazolyl)methanes (*tpzm^R,R'^*), neutral analogues of the more widely studied *tris*–(pyrazolyl)borates (*tp^R,R'^*), are an increasing important class of ligands with a wide variety of coordination and organometallic complexes now reported (Pettinari & Pettinari, 2005). In most of these studies only the simplest members of the series *tpzm* and *tpzm^Me,Me^* which generally form inert sandwich complexes with first row transition metals are utilized (Astley *et al.*, 1993; Reger *et al.*, 2002). In contrast, second generation *tris*–(pyrazolyl)methane ligands (*tpzm^Ph^*, *tpzm^i–Pr^* and *tpzm^t–Bu^*) remain poorly represented owing to their time consuming synthesis and low yields. However, Reger (Reger *et al.*, 2000) recently reported an improved procedure for these ligands, while Fujisawa and co–workers (Fujisawa *et al.*, 2004) have shown that even *tzpm^i–Pr,i–Pr^* may be prepared. Structural studies of *tris*–(pyrazolyl)methanes are even rarer and to date only *tpzm^Me,Me^* has been reported (Declercq & Van Meerssche, 1984; Ochando *et al.*, 1997). Herein, we report the synthesis and the first structural characterization of a second generation *tris*–(pyrazolyl)methane ligand namely, *tpzm^Ph,Me^* (I).

Colourless block shaped crystals of I were grown from CH_2_Cl_2_/*n*–hexane, the compound crystallizing in a monoclinic *Cc* space group. The structure of the molecule I is shown on Fig. 1. The pyrazoles are bonded to the central CH–group in a tetrahedral fashion with N—C1—N angles [112.8 (2)°, 110.4 (2)° and 111.1 (2)°] close to the ideal tetrahedral value of 109.5° and similar to those found in *tpzm^Me,Me^* [110°, 111°, 111° (Declercq & Van Meerssche, 1984)]. In addition, the structure shows that the methyl groups are in the 5–position of the pyrazole rings with the phenyl rings in the 3–position thereby minimizing steric congestion around the central CH–group and confirming the presence of a single regioisomer.

The propeller–like conformation of the molecule can be defined by the angle between the plane formed by H1A, C1 and the first pyrazole N atom and the mean plane of the pyrazole ring. The values for I are 50.4 (2)°, 18.7 (3)° and 36.3 (2)° and are comparable to those observed in the structures of *tpzm^Me,Me^* [the values of each ring averaged over four molecules are 29 (3)°, 23 (2)° and 62 (1)° (Declercq & Van Meerssche, 1984)] and triphenylmethane [30°, 34° and 53°, and 21°, 38° and 47° for each one of the two molecules in the asymmetric unit (Riche & Pascard-Billy, 1974)]. A further method for describing this helical twist is through H1A–C1–N–N torsion angles (Ochando *et al.*, 1997). The torsion angles for I are 133.7 (3)°, -18.3 (4)° and 148.9 (3)° for H1A–C1–N1–N2, H1A–C1–N3–N4 and H1A–C1–N5–N6, respectively. These are in good agreement with the values observed in *tpzm^Me,Me^* [121 (1)°, -21 (1)° and 147 (1)° (Declercq & Van Meerssche, 1984)]. Assuming an α–conformation when the torsion angle is negative and β– when positive, it follows that the conformation in the case of I is β–α–β–, identical to the most stable conformer of *tpzm^Me,Me^* (Declercq & Van Meerssche, 1984).

The pyrazole bond lengths in I vary between 1.328 (3)Å and 1.414 (3)Å and are very similar to those found in *tpzm^Me,Me^* [1.33–1.40Å (Declercq & Van Meerssche, 1984)]. The phenyl rings are essentially co-planar with the pyrazole rings (dihedral angles: 3.4 (1)° and 2.8 (1)°) except in the case of the C20—N5 pyrazole ring in which the dihedral angles between the two planes is 15.7 (2)°.

A further point of interest is the packing within the structure of I which reveals C—H···π interactions between the pyrazole C3—H3 and the centroid of the ring C14/C15/C16/C17/C18/C19 (*Cg*), the phenyl group attached to the α–pyrazole (Fig. 2). All these interactions occur within a single layer of molecules with adjacent layers, which are related by inversion, exhibiting interactions in the opposite direction. Thus, the interactions H3 (*x*+1/2, *y*-1/2, *z*)···*Cg* (*x*, *y*, *z*) and  H3 (*x*-1/2, -*y*+3/2, *z*-1/2)···*Cg*^ii^ (*x*-1, -*y*+1, *z*-1/2) are both 3.202 (2)Å.

### Experimental

Distilled water (20 ml) was added to a 250 ml flask containing a mixture of *Hpz^Ph,Me^* (6.33 g, 40 mmol) and N*Bu*_4_Br (0.68 g, 2 mmol). With vigorous stirring Na_2_CO_3_ (8.5 g, 80 mmol) was added to the reaction mixture. After cooling CHCl_3_ (75 ml) was added and the mixture refluxed for four days yielding a dark yellow–orange emulsion. The mixture was allowed to cool to room temperature and filtered through a Buchner funnel. The organic layer was separated from the aqueous layer, washed with water (3 × 30 ml) and dried over sodium sulfate. The solution was filtered to remove the drying agent and the solvent removed on a rotary evapourator to give a yellow solid. The solid was redissolved in toluene (70 ml) and a catalytic amount of *p*–toluenesulfonic acid (0.1 g, 0.53 mmol) was added. The solution was refluxed for a day giving a yellow solution. The solution was then cooled to room temperature, neutralized with a 5% aqueous Na_2_CO_3_ solution and washed with distilled water (3 × 15 ml). The solution was then dried over sodium sulfate, filtered and the solvent removed on a rotary evapourator resulting in a light brown solid. The solid was dissolved in CH_2_Cl_2_ (20 ml) and chromatographed on a silica gel column that was packed with a CH_2_Cl_2_:toluene (1:1) solution. The fractions containing the desired product were combined and the solvent removed by rotary evapouration to give an off–white solid (1.83 g, 29%). Analysis calculated for C_31_H_28_N_6_: C 76.8, H 5.8, N 17.3%; found: C 76.7, H 5.8, N 17.0%. ESI^+^ MS: (*m/z*) Anal. Calc. 484.60; found: [MH]^+ ^485.65. ^1^H–NMR (CDCl_3_) δ 8.42 (s, 1H, CH), 7.77–7.72 [m, 6H, *o*–H (*Ph*)], 7.38–7.33 [m, 6H, *m*–H (*Ph*)], 7.30–7.28 [m, 3H, *p*–H, (*Ph*)], 6.47 [s, 3H, 4–H (*pz*)] and 2.22 (s, 9H, CH_3_). It should be noted that I (see Fig. 3) was previously reported as a by–product in the synthesis of more complex *tris*–(pyrazolyl)methanes (Goodman & Bateman, 2001). However, it was not isolated and the above represents the first designed synthesis of I.

### Refinement

H atoms were placed geometrically and refined with a riding model (including torsional freedom for methyl groups) and with *U*ĩso~ constrained to be 1.2 (1.5 for CH~3~ groups) times *U*~eq~ of the carrier atom.

The two restraints are generated automatically to prevent the whole structure from wandering in the *a*– and *c*–directions. The 1950 Friedel pairs were merged.

### Crystal data

**Table:**

C_31_H_28_N_6_	*F*_000_ = 1024
*M**_r_* = 484.59	*D*_x_ = 1.249 Mg m^−^^3^
Monoclinic, *C**c*	Mo *K*α radiation λ = 0.71073 Å
Hall symbol: C -2yc	Cell parameters from 879 reflections
*a* = 6.678 (3) Å	θ = 4.6–46.9º
*b* = 21.730 (9) Å	µ = 0.08 mm^−^^1^
*c* = 17.831 (7) Å	*T* = 150 (2) K
β = 94.922 (7)º	Block, colourless
*V* = 2578.2 (19) Å^3^	0.38 × 0.34 × 0.21 mm
*Z* = 4	

### Data collection

**Table:**

Bruker SMART CCD area-detector diffractometer	2932 independent reflections
Radiation source: Fine-focus sealed tube	2129 reflections with *I* > 2σ(*I*)
Monochromator: Graphite	*R*_int_ = 0.039
Detector resolution: 100 pixels mm^-1^	θ_max_ = 27.6º
*T* = 150(2) K	θ_min_ = 1.9º
φ and ω scans	*h* = −8→8
Absorption correction: multi-scan(SADABS; Bruker, 1997)	*k* = −27→25
*T*_min_ = 0.972, *T*_max_ = 0.984	*l* = −19→22
9586 measured reflections	

### Refinement

**Table:**

Refinement on *F*^2^	Secondary atom site location: difference Fourier map
Least-squares matrix: full	Hydrogen site location: inferred from neighbouring sites
*R*[*F*^2^ > 2σ(*F*^2^)] = 0.041	H-atom parameters constrained
*wR*(*F*^2^) = 0.071	*w* = 1/[σ^2^(*F*_o_^2^) + (0.0288*P*)^2^] where *P* = (*F*_o_^2^ + 2*F*_c_^2^)/3
*S* = 0.98	(Δ/σ)_max_ < 0.001
2932 reflections	Δρ_max_ = 0.12 e Å^−^^3^
337 parameters	Δρ_min_ = −0.14 e Å^−^^3^
2 restraints	Extinction correction: none
Primary atom site location: structure-invariant direct methods	

### Special details

**Table:**

Geometry. All s.u.'s (except the s.u. in the dihedral angle between two l.s. planes) are estimated using the full covariance matrix. The cell s.u.'s are taken into account individually in the estimation of s.u.'s in distances, angles and torsion angles; correlations between s.u.'s in cell parameters are only used when they are defined by crystal symmetry. An approximate (isotropic) treatment of cell s.u.'s is used for estimating s.u.'s involving l.s. planes.
Refinement. Refinement of F^2^ against ALL reflections. The weighted R–factor wR and goodness of fit S are based on F^2^, conventional R–factors R are based on F, with F set to zero for negative F^2^. The threshold expression of F^2^ > σ(F^2^) is used only for calculating R–factors(gt) etc. and is not relevant to the choice of reflections for refinement. R–factors based on F^2^ are statistically about twice as large as those based on F, and R–factors based on ALL data will be even larger.

### Fractional atomic coordinates and isotropic or equivalent isotropic displacement parameters (Å^2^)

**Table:**

	*x*	*y*	*z*	*U*_iso_*/*U*_eq_	
N1	0.8396 (3)	0.83570 (9)	0.68651 (11)	0.0408 (5)	
N2	0.6619 (3)	0.85821 (9)	0.65488 (11)	0.0414 (5)	
N3	0.8952 (3)	0.73399 (9)	0.73501 (11)	0.0401 (5)	
N4	1.0565 (3)	0.69625 (9)	0.75166 (11)	0.0399 (5)	
N5	0.7907 (3)	0.74998 (8)	0.60399 (11)	0.0396 (5)	
N6	0.6231 (3)	0.71502 (9)	0.61064 (11)	0.0398 (5)	
C1	0.9043 (4)	0.77355 (10)	0.67027 (14)	0.0420 (6)	
H1A	1.0482	0.7759	0.6589	0.050*	
C2	0.9315 (4)	0.87441 (12)	0.73918 (14)	0.0460 (6)	
C3	0.8081 (4)	0.92398 (12)	0.74119 (15)	0.0493 (7)	
H3A	0.8289	0.9592	0.7724	0.059*	
C4	0.6429 (4)	0.91297 (11)	0.68788 (15)	0.0410 (6)	
C5	0.4662 (4)	0.95123 (10)	0.66896 (14)	0.0424 (6)	
C6	0.3172 (4)	0.93283 (12)	0.61469 (16)	0.0551 (7)	
H6A	0.3321	0.8955	0.5880	0.066*	
C7	0.1474 (5)	0.96829 (13)	0.59919 (17)	0.0646 (8)	
H7A	0.0455	0.9546	0.5625	0.077*	
C8	0.1229 (5)	1.02323 (13)	0.63600 (19)	0.0625 (8)	
H8A	0.0065	1.0477	0.6244	0.075*	
C9	0.2681 (5)	1.04169 (12)	0.68914 (18)	0.0609 (8)	
H9A	0.2528	1.0794	0.7149	0.073*	
C10	0.4385 (4)	1.00619 (12)	0.70633 (16)	0.0548 (7)	
H10A	0.5375	1.0197	0.7442	0.066*	
C11	0.7515 (3)	0.72783 (11)	0.78425 (14)	0.0399 (6)	
C12	0.8255 (3)	0.68485 (11)	0.83497 (14)	0.0407 (6)	
H12A	0.7622	0.6704	0.8773	0.049*	
C13	1.0131 (3)	0.66587 (11)	0.81285 (13)	0.0372 (6)	
C14	1.1513 (4)	0.61970 (11)	0.84821 (14)	0.0396 (6)	
C15	1.0996 (4)	0.58720 (12)	0.91093 (15)	0.0502 (7)	
H15A	0.9766	0.5960	0.9318	0.060*	
C16	1.2253 (5)	0.54219 (12)	0.94321 (17)	0.0597 (8)	
H16A	1.1882	0.5204	0.9861	0.072*	
C17	1.4041 (5)	0.52873 (12)	0.91364 (17)	0.0582 (8)	
H17A	1.4890	0.4972	0.9353	0.070*	
C18	1.4588 (4)	0.56126 (12)	0.85242 (16)	0.0520 (7)	
H18A	1.5836	0.5528	0.8327	0.062*	
C19	1.3338 (4)	0.60609 (11)	0.81947 (15)	0.0443 (6)	
H19A	1.3726	0.6278	0.7768	0.053*	
C20	0.8150 (4)	0.76617 (11)	0.53141 (14)	0.0404 (6)	
C21	0.6590 (4)	0.73918 (10)	0.48918 (14)	0.0412 (6)	
H21A	0.6326	0.7412	0.4360	0.049*	
C22	0.5452 (4)	0.70774 (10)	0.53982 (13)	0.0377 (6)	
C23	0.3596 (4)	0.67181 (10)	0.52414 (14)	0.0394 (6)	
C24	0.2386 (4)	0.65752 (11)	0.58115 (15)	0.0471 (7)	
H24A	0.2784	0.6699	0.6313	0.057*	
C25	0.0610 (4)	0.62552 (12)	0.56614 (18)	0.0548 (7)	
H25A	−0.0209	0.6165	0.6058	0.066*	
C26	0.0024 (4)	0.60663 (12)	0.49386 (19)	0.0565 (8)	
H26A	−0.1195	0.5844	0.4837	0.068*	
C27	0.1197 (4)	0.61980 (12)	0.43680 (18)	0.0589 (8)	
H27A	0.0792	0.6067	0.3870	0.071*	
C28	0.2975 (4)	0.65226 (12)	0.45144 (16)	0.0521 (7)	
H28A	0.3780	0.6613	0.4114	0.063*	
C29	1.1317 (4)	0.86029 (13)	0.77999 (16)	0.0595 (8)	
H29A	1.2324	0.8556	0.7435	0.089*	
H29B	1.1226	0.8220	0.8085	0.089*	
H29C	1.1711	0.8940	0.8146	0.089*	
C30	0.5591 (3)	0.76287 (12)	0.78100 (15)	0.0480 (7)	
H30A	0.4767	0.7475	0.8199	0.072*	
H30B	0.4864	0.7575	0.7313	0.072*	
H30C	0.5879	0.8066	0.7897	0.072*	
C31	0.9840 (4)	0.80561 (11)	0.51083 (16)	0.0507 (7)	
H31A	1.1115	0.7841	0.5231	0.076*	
H31B	0.9839	0.8443	0.5391	0.076*	
H31C	0.9678	0.8144	0.4567	0.076*	

### Atomic displacement parameters (Å^2^)

**Table:**

	*U*^11^	*U*^22^	*U*^33^	*U*^12^	*U*^13^	*U*^23^
N1	0.0423 (12)	0.0441 (11)	0.0359 (12)	0.0008 (10)	0.0025 (9)	−0.0004 (10)
N2	0.0439 (12)	0.0417 (11)	0.0386 (13)	0.0028 (10)	0.0028 (10)	−0.0013 (9)
N3	0.0347 (11)	0.0516 (12)	0.0345 (12)	0.0015 (10)	0.0054 (10)	0.0045 (10)
N4	0.0364 (12)	0.0466 (12)	0.0365 (13)	0.0008 (10)	0.0019 (9)	0.0010 (10)
N5	0.0442 (12)	0.0422 (11)	0.0328 (12)	0.0058 (10)	0.0060 (9)	0.0017 (10)
N6	0.0423 (12)	0.0414 (11)	0.0369 (13)	0.0030 (10)	0.0107 (10)	0.0002 (9)
C1	0.0382 (14)	0.0485 (14)	0.0396 (15)	0.0011 (12)	0.0062 (11)	0.0061 (12)
C2	0.0451 (15)	0.0576 (17)	0.0359 (15)	−0.0132 (13)	0.0060 (12)	−0.0006 (12)
C3	0.0578 (17)	0.0462 (15)	0.0442 (17)	−0.0088 (14)	0.0069 (14)	−0.0074 (12)
C4	0.0465 (16)	0.0419 (14)	0.0361 (15)	−0.0053 (12)	0.0126 (12)	−0.0019 (12)
C5	0.0493 (15)	0.0375 (13)	0.0416 (16)	−0.0019 (12)	0.0107 (13)	0.0032 (12)
C6	0.0687 (19)	0.0474 (15)	0.0473 (18)	0.0085 (15)	−0.0057 (15)	−0.0020 (13)
C7	0.068 (2)	0.0641 (19)	0.060 (2)	0.0108 (17)	−0.0085 (16)	0.0016 (16)
C8	0.0616 (19)	0.0496 (17)	0.078 (2)	0.0107 (15)	0.0150 (17)	0.0142 (16)
C9	0.0630 (19)	0.0450 (16)	0.078 (2)	−0.0001 (15)	0.0269 (17)	−0.0077 (15)
C10	0.0533 (18)	0.0496 (16)	0.063 (2)	−0.0092 (14)	0.0164 (15)	−0.0113 (14)
C11	0.0350 (14)	0.0517 (15)	0.0334 (14)	−0.0044 (12)	0.0053 (11)	−0.0031 (12)
C12	0.0401 (15)	0.0504 (15)	0.0322 (15)	−0.0063 (12)	0.0075 (12)	0.0014 (12)
C13	0.0401 (15)	0.0416 (13)	0.0294 (14)	−0.0063 (11)	−0.0001 (11)	−0.0031 (11)
C14	0.0452 (16)	0.0396 (13)	0.0332 (15)	−0.0083 (11)	−0.0006 (12)	−0.0033 (11)
C15	0.0564 (16)	0.0508 (16)	0.0432 (17)	−0.0055 (14)	0.0030 (14)	0.0046 (14)
C16	0.077 (2)	0.0524 (17)	0.0489 (19)	−0.0083 (16)	−0.0014 (17)	0.0121 (14)
C17	0.076 (2)	0.0380 (15)	0.058 (2)	0.0083 (15)	−0.0095 (17)	−0.0016 (14)
C18	0.0573 (17)	0.0461 (15)	0.0511 (18)	0.0056 (14)	−0.0031 (14)	−0.0081 (14)
C19	0.0487 (15)	0.0450 (14)	0.0385 (15)	−0.0038 (12)	0.0000 (12)	−0.0035 (12)
C20	0.0498 (15)	0.0391 (13)	0.0336 (15)	0.0142 (12)	0.0100 (12)	0.0057 (11)
C21	0.0550 (16)	0.0388 (13)	0.0300 (14)	0.0104 (12)	0.0052 (12)	0.0031 (11)
C22	0.0464 (15)	0.0364 (13)	0.0306 (15)	0.0123 (11)	0.0058 (12)	0.0000 (11)
C23	0.0469 (15)	0.0329 (12)	0.0392 (15)	0.0096 (11)	0.0071 (12)	0.0007 (11)
C24	0.0551 (18)	0.0460 (15)	0.0415 (17)	0.0071 (13)	0.0109 (13)	−0.0008 (13)
C25	0.0552 (18)	0.0478 (15)	0.063 (2)	0.0039 (14)	0.0157 (16)	0.0076 (15)
C26	0.0553 (17)	0.0434 (15)	0.071 (2)	−0.0035 (13)	0.0063 (17)	0.0008 (15)
C27	0.067 (2)	0.0563 (17)	0.052 (2)	−0.0106 (16)	−0.0026 (16)	−0.0054 (14)
C28	0.0645 (19)	0.0527 (16)	0.0399 (16)	−0.0016 (14)	0.0089 (13)	−0.0022 (13)
C29	0.0482 (17)	0.0744 (19)	0.0541 (19)	−0.0096 (15)	−0.0048 (14)	0.0007 (15)
C30	0.0402 (15)	0.0602 (17)	0.0444 (16)	0.0039 (13)	0.0087 (12)	−0.0032 (13)
C31	0.0552 (17)	0.0533 (16)	0.0447 (17)	0.0041 (14)	0.0098 (13)	0.0089 (13)

### Geometric parameters (Å, °)

**Table:**

N1—N2	1.360 (3)	C14—C19	1.394 (4)
N1—C2	1.367 (3)	C15—C16	1.382 (4)
N1—C1	1.455 (3)	C15—H15A	0.9500
N2—C4	1.338 (3)	C16—C17	1.378 (4)
N3—C11	1.361 (3)	C16—H16A	0.9500
N3—N4	1.366 (3)	C17—C18	1.376 (4)
N3—C1	1.445 (3)	C17—H17A	0.9500
N4—C13	1.328 (3)	C18—C19	1.381 (3)
N5—C20	1.364 (3)	C18—H18A	0.9500
N5—N6	1.366 (3)	C19—H19A	0.9500
N5—C1	1.443 (3)	C20—C21	1.364 (3)
N6—C22	1.334 (3)	C20—C31	1.488 (3)
C1—H1A	1.0000	C21—C22	1.406 (3)
C2—C3	1.358 (4)	C21—H21A	0.9500
C2—C29	1.498 (4)	C22—C23	1.471 (3)
C3—C4	1.414 (3)	C23—C24	1.387 (3)
C3—H3A	0.9500	C23—C28	1.393 (4)
C4—C5	1.459 (3)	C24—C25	1.381 (4)
C5—C6	1.386 (3)	C24—H24A	0.9500
C5—C10	1.388 (3)	C25—C26	1.377 (4)
C6—C7	1.379 (4)	C25—H25A	0.9500
C6—H6A	0.9500	C26—C27	1.366 (4)
C7—C8	1.379 (4)	C26—H26A	0.9500
C7—H7A	0.9500	C27—C28	1.387 (4)
C8—C9	1.357 (4)	C27—H27A	0.9500
C8—H8A	0.9500	C28—H28A	0.9500
C9—C10	1.387 (4)	C29—H29A	0.9800
C9—H9A	0.9500	C29—H29B	0.9800
C10—H10A	0.9500	C29—H29C	0.9800
C11—C12	1.363 (3)	C30—H30A	0.9800
C11—C30	1.490 (3)	C30—H30B	0.9800
C12—C13	1.407 (3)	C30—H30C	0.9800
C12—H12A	0.9500	C31—H31A	0.9800
C13—C14	1.468 (3)	C31—H31B	0.9800
C14—C15	1.391 (3)	C31—H31C	0.9800
			
N2—N1—C2	112.83 (19)	C14—C15—H15A	119.6
N2—N1—C1	120.99 (19)	C17—C16—C15	120.4 (3)
C2—N1—C1	125.8 (2)	C17—C16—H16A	119.8
C4—N2—N1	104.46 (19)	C15—C16—H16A	119.8
C11—N3—N4	112.77 (19)	C18—C17—C16	119.5 (3)
C11—N3—C1	130.86 (19)	C18—C17—H17A	120.2
N4—N3—C1	116.37 (19)	C16—C17—H17A	120.2
C13—N4—N3	104.67 (19)	C17—C18—C19	120.5 (3)
C20—N5—N6	113.01 (19)	C17—C18—H18A	119.7
C20—N5—C1	126.1 (2)	C19—C18—H18A	119.7
N6—N5—C1	120.23 (19)	C18—C19—C14	120.6 (3)
C22—N6—N5	103.87 (19)	C18—C19—H19A	119.7
N5—C1—N3	112.83 (19)	C14—C19—H19A	119.7
N5—C1—N1	110.38 (18)	N5—C20—C21	105.4 (2)
N3—C1—N1	111.1 (2)	N5—C20—C31	122.5 (2)
N5—C1—H1A	107.4	C21—C20—C31	132.1 (2)
N3—C1—H1A	107.4	C20—C21—C22	106.5 (2)
N1—C1—H1A	107.4	C20—C21—H21A	126.8
C3—C2—N1	105.6 (2)	C22—C21—H21A	126.8
C3—C2—C29	131.9 (3)	N6—C22—C21	111.2 (2)
N1—C2—C29	122.5 (3)	N6—C22—C23	119.8 (2)
C2—C3—C4	106.8 (2)	C21—C22—C23	129.0 (2)
C2—C3—H3A	126.6	C24—C23—C28	117.9 (2)
C4—C3—H3A	126.6	C24—C23—C22	120.9 (2)
N2—C4—C3	110.4 (2)	C28—C23—C22	121.1 (2)
N2—C4—C5	120.7 (2)	C25—C24—C23	121.0 (3)
C3—C4—C5	128.9 (2)	C25—C24—H24A	119.5
C6—C5—C10	117.8 (3)	C23—C24—H24A	119.5
C6—C5—C4	121.0 (2)	C26—C25—C24	120.1 (3)
C10—C5—C4	121.2 (2)	C26—C25—H25A	119.9
C7—C6—C5	120.5 (3)	C24—C25—H25A	119.9
C7—C6—H6A	119.7	C27—C26—C25	120.0 (3)
C5—C6—H6A	119.7	C27—C26—H26A	120.0
C6—C7—C8	121.1 (3)	C25—C26—H26A	120.0
C6—C7—H7A	119.4	C26—C27—C28	120.2 (3)
C8—C7—H7A	119.4	C26—C27—H27A	119.9
C9—C8—C7	118.8 (3)	C28—C27—H27A	119.9
C9—C8—H8A	120.6	C27—C28—C23	120.8 (3)
C7—C8—H8A	120.6	C27—C28—H28A	119.6
C8—C9—C10	120.9 (3)	C23—C28—H28A	119.6
C8—C9—H9A	119.6	C2—C29—H29A	109.5
C10—C9—H9A	119.6	C2—C29—H29B	109.5
C9—C10—C5	120.9 (3)	H29A—C29—H29B	109.5
C9—C10—H10A	119.6	C2—C29—H29C	109.5
C5—C10—H10A	119.6	H29A—C29—H29C	109.5
N3—C11—C12	105.1 (2)	H29B—C29—H29C	109.5
N3—C11—C30	125.3 (2)	C11—C30—H30A	109.5
C12—C11—C30	129.6 (2)	C11—C30—H30B	109.5
C11—C12—C13	107.2 (2)	H30A—C30—H30B	109.5
C11—C12—H12A	126.4	C11—C30—H30C	109.5
C13—C12—H12A	126.4	H30A—C30—H30C	109.5
N4—C13—C12	110.3 (2)	H30B—C30—H30C	109.5
N4—C13—C14	121.3 (2)	C20—C31—H31A	109.5
C12—C13—C14	128.4 (2)	C20—C31—H31B	109.5
C15—C14—C19	118.2 (2)	H31A—C31—H31B	109.5
C15—C14—C13	120.2 (2)	C20—C31—H31C	109.5
C19—C14—C13	121.6 (2)	H31A—C31—H31C	109.5
C16—C15—C14	120.7 (3)	H31B—C31—H31C	109.5
C16—C15—H15A	119.6		
